# An Investigation of the Relationship Between Social Support, Psychological Resilience, and Post‐Traumatic Growth in Patients With Epilepsy

**DOI:** 10.1002/brb3.71721

**Published:** 2026-09-23

**Authors:** Ercan Bakir, Gülcan Bahçecioğlu Turan, Zülfünaz Özer

**Affiliations:** ^1^ Faculty of Health Sciences, Nursing Department Adıyaman University Adıyaman Turkey; ^2^ Faculty of Health Sciences, Nursing Department Fırat University Elazığ Turkey; ^3^ Department of Nursing, Faculty of Health Sciences Istanbul Sabahattin Zaim University Istanbul Turkey

**Keywords:** epilepsy, mediation analysis, post‐traumatic growth, psychological resilience, social support

## Abstract

**Objective:**

This study aimed to examine the associations among perceived social support, psychological resilience, and post‐traumatic growth (PTG) in patients with epilepsy and to explore whether psychological resilience statistically mediated the association between social support and PTG.

**Method:**

This cross‐sectional correlational study was conducted with 158 patients with epilepsy recruited through snowball sampling between May and December 2025. Data were collected using the personal information form, the Multidimensional Scale of Perceived Social Support, the Brief Resilience Scale, and the post‐traumatic growth inventory. Descriptive statistics, Pearson correlation analysis, hierarchical regression analysis, and PROCESS Macro (Model 4) were used for data analysis.

**Results:**

Significant positive correlations were found between social support and psychological resilience (*r* = 0.759), social support and PTG (*r* = 0.491), and psychological resilience and PTG (*r* = 0.465) (*p* < 0.001). Hierarchical regression analysis showed that social support was positively associated with PTG (*β* = 0.462, *p* < 0.001), whereas the direct association between psychological resilience and PTG was not statistically significant (*β* = 0.168, *p* = 0.120). Bootstrapping analysis indicated a significant indirect association of psychological resilience in the relationship between social support and PTG (indirect effect = 0.220; 95% CI [0.007−0.466]).

**Conclusion:**

Perceived social support, psychological resilience, and post‐traumatic growth were positively associated in patients with epilepsy. The findings suggest that psychological resilience may play a role in the observed association between social support and post‐traumatic growth. Given the cross‐sectional nature of the study, longitudinal research is needed to clarify the direction and temporal nature of these relationships.

## Graphical abstract

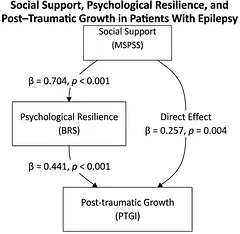

## 1 Introduction

Epilepsy is a chronic neurological disorder characterized by recurrent seizures caused by abnormal and excessive neuronal activity in the central nervous system (Beniczky et al. 2025). It affects more than 70 million people worldwide and is recognized as a major global neurological health burden (Organization 2023; Winter et al. 2022). Beyond its clinical manifestations, epilepsy is associated with substantial psychosocial challenges, including uncertainty about seizure occurrence, perceived loss of control, and stigma, all of which may negatively affect quality of life and psychological well‐being (Karaca and Durna 2018; Walker and Peterson 2024). Living with epilepsy requires continuous adaptation and coping with both medical and social demands.

In this context, social support is considered an important external resource that may help individuals manage stress. Social support, provided by family, friends, and healthcare professionals, includes emotional, informational, and instrumental assistance (Yang et al. 2024). In individuals with epilepsy, higher perceived social support has been associated with better treatment adherence, lower psychological distress, and reduced stigma‐related burden (Düken and Belli 2025).

In addition to external resources, internal psychological factors are also important in adaptation to chronic illness. Psychological resilience refers to the ability to maintain or regain psychological well‐being in the face of adversity, including unpredictable seizures and social challenges (Fu et al. 2025). In epilepsy populations, resilience has been associated with better psychological adjustment and improved quality of life (Fu et al. 2025; Tedrus et al. 2020).

Despite the challenges of epilepsy, some individuals may also experience positive psychological changes following adverse experiences. Post‐traumatic growth (PTG) refers to positive changes such as improved interpersonal relationships, changed life priorities, and enhanced personal strength following trauma (Turan et al. 2023). In epilepsy, traumatic experiences may arise from seizure‐related injuries, unpredictability, and social stigma (Pepi et al. 2024). Previous studies suggest that both social support and psychological resilience are positively associated with PTG in chronic illness populations, including epilepsy (Salmanipour et al. 2025).

Recent developments in PTG research suggest that positive and negative psychological changes following adversity may coexist and should be considered as related yet distinct outcomes of trauma (Cann et al. 2011; Kroemeke et al. 2017; Pięta and Rzeszutek 2023). In this context, posttraumatic depreciation (PTD) refers to negative changes occurring in the same domains as PTG, such as deterioration in interpersonal relationships, reduced personal strength, and diminished appreciation of life (Cann et al. 2011). PTG and PTD are considered relatively independent processes, and individuals may experience both simultaneously (Kroemeke et al. 2017; Pięta and Rzeszutek 2023).

Despite the growing body of research on PTG in chronic illness populations, studies examining psychosocial adaptation in epilepsy remain limited. Existing research has primarily focused on pairwise associations—such as social support and PTG (Ma et al. 2022; Salmanipour et al. 2025), or resilience and PTG (C. Li et al. 2021; Seiler and Jenewein 2019)—without integrating these variables within a unified mediational framework. Furthermore, although several studies have investigated epilepsy‐related psychosocial outcomes in Turkey (Düken and Belli 2025; Karakaş et al. 2022; Turan et al. 2023), no previous research has specifically examined the mediating role of psychological resilience in the relationship between perceived social support and PTG in this population. Given the cultural and healthcare context of Turkey, where previous studies have suggested that family‐centered support systems may play an important role (Düken and Belli 2025; Karakaş et al. 2022), understanding the associations between social support and positive psychological outcomes may have important implications for culturally sensitive intervention development.

**FIGURE 1:**
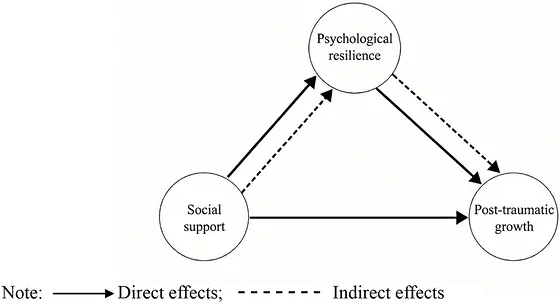
Proposed model.

Therefore, this study aimed to examine the relationships among perceived social support, psychological resilience, and PTG in individuals with epilepsy, and to investigate whether psychological resilience is associated with the relationship between perceived social support and PTG. This study is expected to contribute to the literature by clarifying the relationships among these variables in adaptation to epilepsy and by informing future psychosocial intervention approaches. The proposed study model is presented in Figure 1.

### 1.1 Research Questions

1. What are the levels of social support, psychological resilience, and PTG in patients with epilepsy?
2. Is there a relationship between social support and psychological resilience in patients with epilepsy?
3. Is there a relationship between social support and PTG in patients with epilepsy?
4. Is there a relationship between psychological resilience and PTG in patients with epilepsy?
5. Does psychological resilience play a mediating role in the relationship between social support and PTG in patients with epilepsy?

## 2 Methods

### 2.1 Design

This study was a descriptive correlational study designed to examine the relationships among the variables and to test the mediating role of psychological resilience in the relationship between social support and PTG.

### 2.2 Participants and sample

The study population consisted of individuals diagnosed with epilepsy who were reached through snowball sampling using data collection forms created via Google Docs between May and December 2025. A priori power analysis was performed using the G*Power 3.1.9.4 software to determine the number of patients to be included in the study. Since no similar study could be found in the literature, an effect size of moderate magnitude (*f* ^2^ = 0.15) was adopted for the multiple regression analysis, based on Cohen's effect size table. Assuming a 95% confidence interval, a significance level of 0.05, and a statistical power of 0.95, the minimum sample size required for the study was determined to be 74 participants. Data collection forms created using Google Docs were sent via email and social media channels to individuals diagnosed with epilepsy across Turkey; participants were asked to fill out the form and share it with others in their network who have been diagnosed with epilepsy. A total of 360 people accessed the online survey form. However, because 26 people did not meet the inclusion criteria (being 18 years of age or older, having been diagnosed with epilepsy for at least 6 months, and completing the survey within 5–10 min) and 176 people did not consent to participate in the survey, the study was completed with 158 participants.

### 2.3 Measurement

Data were collected using the Personal Information Form and three standardized scales: the Multidimensional Scale of Perceived Social Support (MPSSS), the Brief Resilience Scale (BRS), and the PTG inventory (PTGI).

#### 2.3.1 Personal Information Form

This form, prepared by the researchers, included sociodemographic characteristics (gender, age, marital status, place of residence, educational background, and employment status) and disease‐related characteristics (duration of illness, seizure frequency, seizure type, and presence of other chronic conditions) (Düken and Belli 2025).

#### 2.3.2 Multidimensional Perceived Social Support Scale (MPSSS)

The MPSSS is a 12‐item, 7‐point Likert‐type scale assessing perceived support from family, friends, and significant others. Total scores range from 12 to 84, with higher scores indicating greater perceived social support (Zimet et al. 1988). The Turkish adaptation demonstrated good internal consistency (Eker 2001), with a Cronbach's alpha coefficient of 0.89. In the present study, the Cronbach's alpha coefficient was 0.92.

#### 2.3.3 Brief Resilience Scale (BRS)

The BRS (Smith et al. 2008) is a 6‐item, 5‐point Likert‐type scale assessing psychological resilience. Total scores range from 6 to 30, with higher scores indicating greater resilience. The Turkish adaptation demonstrated acceptable internal consistency (Doğan 2015; Smith et al. 2008). While the Cronbach's alpha coefficient for the Turkish adaptation of the scale was 0.79 (Doğan 2015), it was found to be 0.78 in this study.

#### 2.3.4 Post‐Traumatic Growth Inventory (PTGI)

The PTGI is a 21‐item, 6‐point Likert‐type scale assessing positive changes following trauma across five domains: relationships with others, new opportunities, personal strength, spiritual change, and appreciation of life. Total scores range from 0 to 105, with higher scores indicating greater PTG (Tedeschi and Calhoun 1996). In the Turkish adaptation of the scale, the Cronbach's alpha coefficient was reported as 0.92 (Kağan et al. 2012). In the present study, the Cronbach's alpha coefficient was 0.81.

### 2.4 The Ethical Aspects of the Research

Permission to conduct this study was obtained from the Ethics Committee of Istanbul Sabahattin Zaim University (Decision no. 2025/01). The study was conducted in accordance with the principles of the Helsinki Declaration on Human Rights. The researchers explained the purpose and procedure of the study to the patients and obtained their written informed consent.

### 2.5 Data Analysis

Data were analyzed using SPSS version 26.0. Normality was assessed by examining skewness and kurtosis values; values within the range of −1.5 to +1.5 were considered acceptable for a normal distribution. Descriptive statistics and Pearson correlation analysis were applied.

The mediating relationships between MPSSS, BRS, and PTGI scores were tested using Model 4 of the PROCESS Macro (Hayes) with 5000 bootstrap resamples to evaluate indirect effects. Hierarchical regression analysis was used to examine the additional contribution of each variable block to the dependent variable. Based on the literature, age, gender, educational status, year of diagnosis, and disease severity were included as control variables in the first block; categorical variables (gender, educational status) were dummy‐coded. Social support was added in the second block, and psychological resilience in the third block, to examine their predictive effects on PTG. A p‐value of < 0.05 was considered statistically significant.

## 3 Results

The study included 158 participants. Most of the participants—60.1%—are women, 65.2% are married, 32.9% have a high school diploma, and 55.7% are employed. 65.2% of participants have incomes equal to their expenses, and 84.2% live in a nuclear family.

The duration of diagnosis is most commonly 1–3 years (41.8%). Over the past year, two seizures have been reported in 31.6% of cases, and the most common type of seizure is one with an unknown onset (58.9%). 72.8% of participants are taking antiepileptic medications, and the most common treatment regimen involves the use of multiple medications (44.3%). The mean age of participants was 37.49 ± 9.02 years, with an age range of 20–57 years (Table 1).

**TABLE 1:** Descriptive characteristics of the patients.

Characteristics	Number (*n* = 158)	%
**Gender** Female Male	95 63	60.1 39.9
**Marital status** Married Single	103 55	65.2 34.8
**Educational status** Literate Elementary school High school Bachelor's degree or higher	13 48 52 45	8.2 30.4 32.9 28.5
**Working status** Yes No	88 70	55.7 44.3
**Income status** Income<expense Income = expense Income>expense	41 103 14	25.9 65.2 8.9
**Family structure** Nuclear family Extended family	133 25	84.2 15.8
Duration of diagnosis 6–12 months 1–3 years 2–5 years 5 years or more	47 66 24 21	29.7 41.8 15.2 13.3
**Number of seizures in the past year** **None** 1 2 3 4 or more	30 35 50 32 11	19.0 22.2 31.6 20.3 7.0
**Seizure type** Focal start Unknown start Generalized start	47 93 18	29.7 58.9 11.4
**Presence of another chronic disease** Yes No	54 104	34.2 65.8
**Antiseizure medication use** Yes No	115 43	72.8 27.2
**Number of antiseizure medications used** I use only one medication I use more than one I do not use any	45 70 43	28.5 44.3 27.2
	**X ± SD**	**Min‐Max**
**Age**	37.49 ± 9.02	20–57

*Note*: **X = mean; SD = standard deviation.

The mean scores for the participants on the MSPSS scale were 54.18 ± 14.32, on the BRS scale were 19.69 ± 4.78, and on the PTGI scale were 49.94 ± 18.28. The skewness and kurtosis values of the scales fall within the limits of a normal distribution. The internal consistency coefficients for the scales were found to be 0.92 for the MSPSS, 0.78 for the BRS, and 0.81 for the PTGI. According to the correlation analysis, there is a strong positive correlation between the MSPSS and the BRS (*r* = 0.735). There is a moderate positive correlation between the MSPSS and the PTGI (*r* = 0.594). A moderate positive correlation was also found between the BRS and the PTGI (*r* = 0.654). All correlations are statistically significant (*p* < 0.01). (Table 2)

**TABLE 2:** Correlation coefficients of MSPSS, BRS, and PTGI.

Scales	Mean ± sd	Skew.	Kurt.	a	1	2	3
**1**. MSPSS	54.18 ± 14.32	0.086	1.097	0.92	1		
**2**. BRS	19.69 ± 4.78	−0.140	1.596	0.78	0.735**	1	
**3**. PTGI	49.94 ± 18.28	0.385	1.738	0.81	0.594**	0.654**	1

*Note*: **Correlation is significant at the 0.01 level; a = Cronbach's alpha

Abbreviations: BRS = brief resilience scale; MSPSS = multidimensional perceived social support scale; PTGI = post‐traumatic growth inventory.

The results of the hierarchical regression analysis conducted to determine the predictors of participants’ PTG are presented in Table 3. All three models constructed within the scope of the analysis were found to be statistically significant (*p* < 0.05). In Model 1, participants’ demographic characteristics (age, gender, educational level, duration of illness, and annual seizure frequency) were included in the analysis. The results indicated that age, gender, education, and duration of illness did not have a significant effect on PTG (*p* > 0.05). However, having 4 (*β* = 0.236; *p* = 0.036) and 5 (*β* = 0.265; *p* = 0.005) seizures in the past year significantly and positively predicted PTG. This initial model explained 13.4% of the total variance (*R* ^2^ = 0.134; Adj. *R* ^2^ = 0.062; *p* = 0.042). In Model 2, the variable “social support” was added to the analysis. With the inclusion of social support, there was a significant increase of 28.1% in the explained variance (Δ*R* ^2^ = 0.281; Δ*F* = 69.234, *p* < 0.001). At this stage, social support (*β* = 0.570; *p* < 0.001) emerged as the strongest predictor of PTG. With the inclusion of social support, the previously significant effects of seizure frequency variables became non‐significant (*p* > 0.05). In Model 3, “psychological resilience” was included in the analysis. The addition of psychological resilience contributed an additional 7.7% to the explanatory power of the model (Δ*R* ^2^ = 0.077; Δ*F* = 21.561, *p* < 0.001). In the final model, both psychological resilience (*β* = 0.434; *p* < 0.001) and social support (*β* = 0.261; *p* = 0.005) were found to significantly and positively predict PTG. The final model explained 49.2% of the total variance in PTG (*R* ^2^ = 0.492; Adj. *R* ^2^ = 0.442; *p* < 0.001). The results of the path analysis examining the associations between social support, psychological resilience, and PTG are presented in Table 4.

**TABLE 3:** Hierarchical regression analysis predicting posttraumatic growth.

Model	Independent variables	Unstandardized coefficients		Standardized coefficients	t	Sig.	95% CI	
		B	S.E	β			Lower bound	Upper bound
Model 1	Number of seizures in the last year (4)	10.712	5.074	0.236	2.111	0.036	0.683	20.741
	Number of seizures in the last year (5)	18.999	6.683	0.265	2.843	0.005*	5.790	32.209
Model 2	Social support (MSPSS)	0.728	0.087	0.570	8.321	0.000	0.555	0.901
Model 3	Social support (MSPSS)	0.333	0.118	0.261	2.828	0.005	0.100	0.567
	Psychological resilience (BRS)	1.659	0.357	0.434	4.463	0.000	0.953	2.365
**Model 1**: *R* ^2^ = 0.134; Adj.*R* ^2^ = 0.062; *p* = 0.042								
**Model 2**: *R* ^2^ = 0.415; Adj. *R* ^2^ = 0.362; Δ*R* ^2^ = 0.281; *p* < 0.001								
**Model 3**: *R* ^2^ = 0.492; Adj. *R* ^2^ = 0.442; Δ*R* ^2^ = 0.077; *p*< 0.001								

*Note*: Dependent variable = posttraumatic growth; seizure frequency categories were dummy coded, with “4 seizures” and “5 or more seizures in the past year” entered as indicator variables. ∗Significance level was accepted as *p* < 0.05. CI, confidence interval; SE, standard error; β, standardized regression coefficient; *R* ^2^ = coefficient of determination; Adj *R* ^2^ = adjusted coefficient of determination; Δ*R* ^2^ = change in *R* ^2^.

**TABLE 4:** Results of the path analysis between social support, psychological resilience, and post‐traumatic growth.

					95% CI			
Specific direct effect	b	S.E.	*t*	*p*	Lower	Upper	β	*R* ^2^
Social support→ psychological resilience	0.235	0.019	12.379	0.000	0.197	0.273	0.704	0.557
Psychological resilience → post‐traumatic growth	1.685	0.339	4.961	0.000	0.952	2.365	0.441	
Social support →post‐traumatic growth	0.328	0.112	2.911	0.004	0.105	0.551	0.257	
**Specific indirect effect**								
Social support → psychological resilience → post‐traumatic growth	0.396	0.087	—	—	0.218	0.566	0.310	
**Total effect**								
Social support → post‐traumatic growth	0.725	0.085	8.473	0.000	0.556	0.894	0.568	0.386

*Note*: Seizure frequency was included in the model as a control variable (covariate). b = regression coefficient; S.E. = standard error; β = standardized coefficient; CI = 95% confidence interval.

**FIGURE 2:**
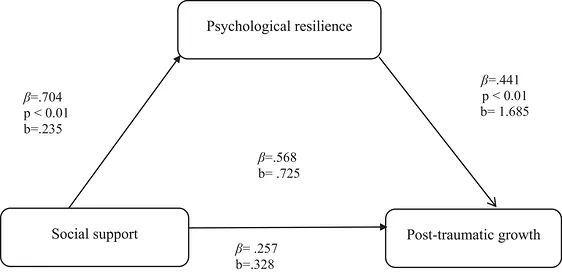
Path analysis.

The path analysis model examining the associations between social support, psychological resilience, and PTG is presented in Figure 2. The results indicated that social support was significantly associated with psychological resilience (*b* = 0.235, S.E. = 0.019, *t* = 12.379, *p* < 0.001, *β* = 0.704, *R* ^2^ = 0.557), suggesting that individuals with higher levels of social support tend to report greater psychological resilience.

Psychological resilience was also significantly associated with PTG (*b* = 1.685, S.E. = 0.339, *t* = 4.961, *p* < 0.001, *β* = 0.441). In addition, social support showed a significant direct association with PTG (*b* = 0.328, S.E. = 0.112, *t* = 2.911, *p* = 0.004, *β* = 0.257), as well as an indirect effect through psychological resilience (*b* = 0.396, S.E. = 0.087, 95% CI = 0.218–0.566, *β* = 0.310). The bootstrap confidence interval indicated that the indirect effect was statistically significant. Overall, the model explained 55.7% of the variance in psychological resilience and 38.6% of the variance in PTG. Indirect effects were estimated using 5000 bootstrap samples, and statistical significance was determined based on 95% confidence intervals not crossing zero.

## 4 Discussion

This study examined perceived social support, psychological resilience, and PTG among individuals with epilepsy, as well as the associations among these variables.

The findings indicated a moderate level of perceived social support, consistent with previous studies reporting similar levels in epilepsy populations (Düken and Belli 2025; Karakaş et al. 2022; Liu et al. 2024). This pattern may reflect the dual nature of social experiences in epilepsy, where strong family‐based support is often present, while broader social networks may be limited due to stigma, employment challenges, and social withdrawal (Alkoblan et al. 2023; Evett et al. 2021). In the Turkish context, where previous studies have highlighted the importance of family‐centered support systems (Düken and Belli 2025; Karakaş et al. 2022), family members may play an important role in providing practical and psychosocial support throughout the course of epilepsy management, which may contribute to patients’ perceived social support.

Psychological resilience was also moderate, aligning with previous findings in epilepsy populations (K. Li et al. 2025; Tedrus et al. 2020). Resilience, as a dynamic capacity to adapt to chronic stressors such as seizure unpredictability, stigma, and functional limitations (Fu et al. 2025; Smith et al. 2008), may be shaped by both illness‐related burden and available psychosocial resources. The present findings suggest that individuals with epilepsy maintain a moderate level of psychological adaptation despite ongoing disease‐related challenges.

PTG was similarly at a moderate level, indicating that individuals with epilepsy may experience positive psychological changes alongside illness‐related distress. This finding is consistent with prior research demonstrating that chronic illness experiences can involve both psychological suffering and positive reappraisal (Turan et al. 2023; Yıldırım et al. 2023). In epilepsy, where individuals often face sudden seizures, physical risk, and social stigma (Pepi et al. 2024), the presence of PTG may suggest that individuals with epilepsy can report positive psychological changes alongside ongoing illness‐related challenges and distress.

A strong positive association was found between perceived social support and resilience, suggesting that individuals with higher perceived social support tend to report higher resilience levels. This finding is consistent with previous literature highlighting social support as an important psychosocial resource associated with adaptive coping and psychological adjustment in chronic illness populations (Wang and Liu 2025; Yıldırım et al. 2023; Zhao et al. 2025). Within this framework, social relationships may serve as external resources that are linked with individuals’ capacity to manage stress and maintain psychological stability.

A moderate positive association was observed between perceived social support and PTG, consistent with PTG theory emphasizing the role of interpersonal support in facilitating cognitive processing and meaning reconstruction after stressful life events (Ma et al. 2022; Tedeschi and Calhoun 2004). In epilepsy, supportive environments may be associated with more adaptive interpretations of illness‐related experiences.

Similarly, resilience showed a moderate positive association with PTG, aligning with studies identifying resilience as a key correlate of PTG in chronic illness populations (J. Li et al. 2020; Seiler and Jenewein 2019). This relationship may indicate that resilience and PTG tend to co‐occur among individuals facing long‐term illness‐related challenges.

Path analysis indicated that psychological resilience statistically mediated the association between perceived social support and PTG. This finding is consistent with a statistical mediation model in which psychological resilience was associated with the link between social support and PTG. In other words, higher perceived social support was associated with higher resilience, and both variables were positively associated with PTG. This finding is consistent with theoretical models proposing that social resources are associated with psychological adaptation processes linked with PTG (Fletcher and Sarkar 2013; Tedeschi and Calhoun 2004).

At the same time, recent studies have suggested that PTG and PTD may coexist as distinct responses to adversity rather than representing opposite ends of a single continuum (Cann et al. 2011; Kroemeke et al. 2017; Pięta and Rzeszutek 2023). Therefore, the present findings should be interpreted within a broader framework acknowledging that PTG and PTD may coexist following illness‐related challenges and should not be considered opposite ends of a single continuum. Future research examining both PTG and PTD simultaneously could provide a more comprehensive understanding of post‐trauma outcomes in this population.

The finding that social support explained 55% of the variance in resilience highlights the strong statistical association between these variables and the close relationship between external social resources and psychological adaptation in epilepsy.

From a clinical perspective, these findings suggest that strengthening both external social resources and internal psychological capacities may be relevant to psychological adaptation in epilepsy. However, given the cross‐sectional design, these implications should be considered preliminary and hypothesis‐generating.

### 4.1 Limitations of the Study

As with all studies, this study also has certain limitations that need to be addressed. First, this was a cross‐sectional study. Therefore, causality cannot be directly inferred. Longitudinal studies are needed to clarify the direction and stability of these relationships over time. Second, the use of snowball sampling and online data collection may have introduced selection bias, as participants were recruited via email and social media, which may have resulted in a sample with higher digital literacy and potentially different sociodemographic characteristics compared to the broader epilepsy population in Turkey. Furthermore, the high rate of non‐consent (176 of 360 individuals) may limit the generalizability of findings. Third, since all the measurement tools used in this study are self‐report measures, participants may have overestimated or underestimated their levels of psychological resilience, perceived social support, and PTG. Additionally, clinical information such as the duration of diagnosis and seizure frequency was based on patient self‐reports rather than medical records, which may introduce recall bias. Fourth, the study sample consists of epilepsy patients being treated at a specific healthcare facility and the specific cultural context of Turkey, which may limit the generalizability of the findings to all epilepsy patients. Furthermore, while this study controlled for several socio‐demographic and clinical variables (e.g., age, gender, and seizure frequency), other potential confounders such as personality traits, pre‐existing psychiatric comorbidities, or specific coping styles were not accounted for, which might also influence the levels of PTG.

Finally, PTG was assessed using a self‐report measure (PTGI). Previous studies have noted that PTGI scores may reflect perceived growth rather than actual psychological change (Jayawickreme and Blackie 2014); therefore, the findings should be interpreted with caution.

## 5 Conclusions and Recommendations

This study found that individuals with epilepsy reported moderate levels of perceived social support, psychological resilience, and PTG, with significant positive associations among these variables.

Path analysis indicated that perceived social support was positively associated with psychological resilience, which was in turn associated with PTG. The results further suggest that psychological resilience may statistically mediate the relationship between perceived social support and PTG. These findings highlight the interconnected relationships among external social resources, internal psychological capacity, and positive psychological outcomes in individuals with epilepsy.

From a clinical perspective, the findings suggest that epilepsy care should extend beyond seizure management to include routine assessment of psychosocial well‐being. Interventions aimed at strengthening perceived social support and psychological resilience may be beneficial in supporting patients’ psychological adjustment. In particular, involving family members in supportive care strategies, particularly in contexts where family support plays an important role, may help enhance patients' psychosocial resources.

Future research should use longitudinal designs to clarify better how perceived social support, psychological resilience, and PTG develop and interact over time. This would provide stronger evidence regarding the temporal ordering and stability of these relationships in epilepsy populations. Additionally, studies examining both PTG and PTD simultaneously could provide a more comprehensive understanding of post‐trauma outcomes in this population. Cross‐cultural studies are also needed to determine the generalizability of these findings across different healthcare and cultural settings.

## Author Contributions

All listed authors meet the authorship criteria, and all authors agree with the content of the manuscript.

## Funding

The authors have nothing to report.

## Conflicts of Interest

The authors declare no conflicts of interest.

## Data Availability

The datasets generated and/or analyzed during the current study are not publicly available due to ethical and privacy restrictions; however, they are available from the corresponding author on reasonable request and with appropriate institutional permissions.
